# Unilateral malignant optic glioma following glioblastoma multiforme in the young: a case report and literature review

**DOI:** 10.1186/s12886-017-0415-5

**Published:** 2017-03-11

**Authors:** Chia-Ying Lin, Hsiu-Mei Huang

**Affiliations:** Department of Ophthalmology, Kaohsiung Chang Gung Memorial Hospital and Chang Gung University College of Medicine, 123 Ta-Pei Road, Kaohsiung, Taiwan

**Keywords:** Optic disc edema, Malignant optic glioma, Glioblastoma multiforme, Young

## Abstract

**Background:**

Malignant optic gliomas are rare, but they rapidly become lethal visual pathway tumors. We present the clinical course, treatment, and prognosis of a case of unilateral malignant optic glioma in a young man with a history of brain glioblastoma multiforme (GBM).

**Case presentation:**

A 21-year-old man, who had GBM 7 years ago complained of a transient shadow in his vision and presented with normal visual acuity but optic disc edema and an enlarged blind spot in the right eye (oculus dexter, OD). Magnetic resonance imaging (MRI) showed a right intraorbital optic nerve tumor without a brain lesion. Chiasm involvement and severe vision deterioration occurred 3 months later. A biopsy of the right optic nerve revealed glioblastoma. Concurrent chemoradiotherapy (CCRT) prevented involvement of the fellow eye 1 year after symptom onset.

**Conclusion:**

This report demonstrated that a regular ocular exam should be recommended for several years after GBM. In young healthy patients who are able to undergo chemotherapy and radiotherapy, visual function in the fellow eye can be preserved.

## Background

Glioblastoma multiforme (GBM) is the most frequent and lethal type of malignant primary brain tumor in humans, and it has the poorest prognosis with a median survival of generally < 1 year from the time of diagnosis [1–3]. GBM is rarely observed in children, which comprising only 5–10% of childhood intracranial neoplasms [4].

Malignant optic glioma is rare and was first defined by Hoyt et al. in 1973 [5]. It is a high grade astrocytoma, presenting as an anaplastic astrocytoma (AA) (WHO grade III) or glioblastoma (WHO grade IV) [6]. Patients frequently have bilateral visual loss within a few weeks and die within 1–2 years [7, 8]. Here, we report a young man presenting with unilateral malignant optic glioma after having GBM for >5 years.

## Case presentation

**Fig. 1:**
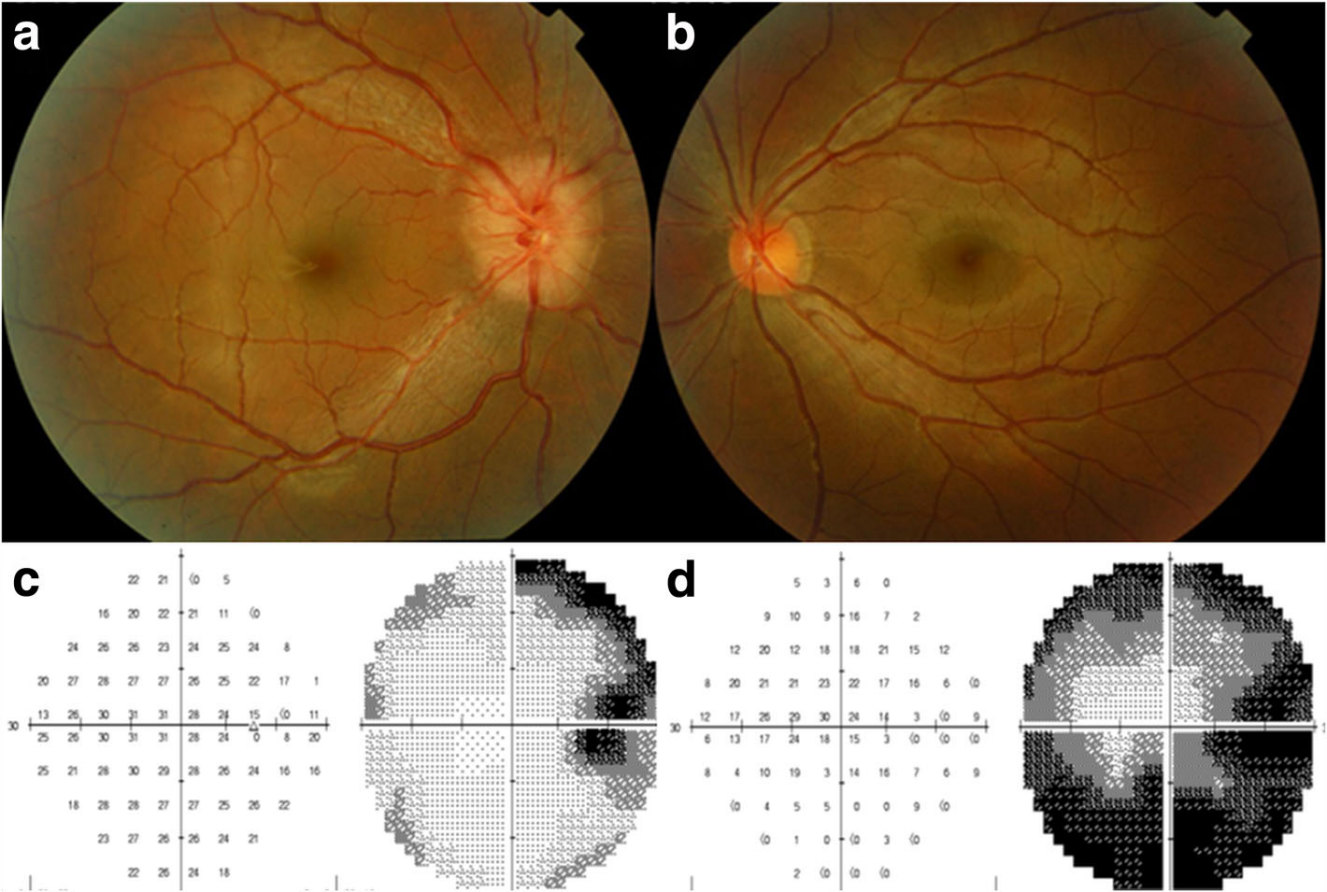
Fundus imaging showed (**a**) a swollen optic disc in the right eye, (**b**) a normal optic disc in the left eye. Humphrey automated perimetry (SITA) revealed (**c**) an enlarged blind spot in the right eye at the initial symptom onset and (**d**) severe constriction with only central area preservation in the right eye 3 months after symptom onset

**Fig. 2:**
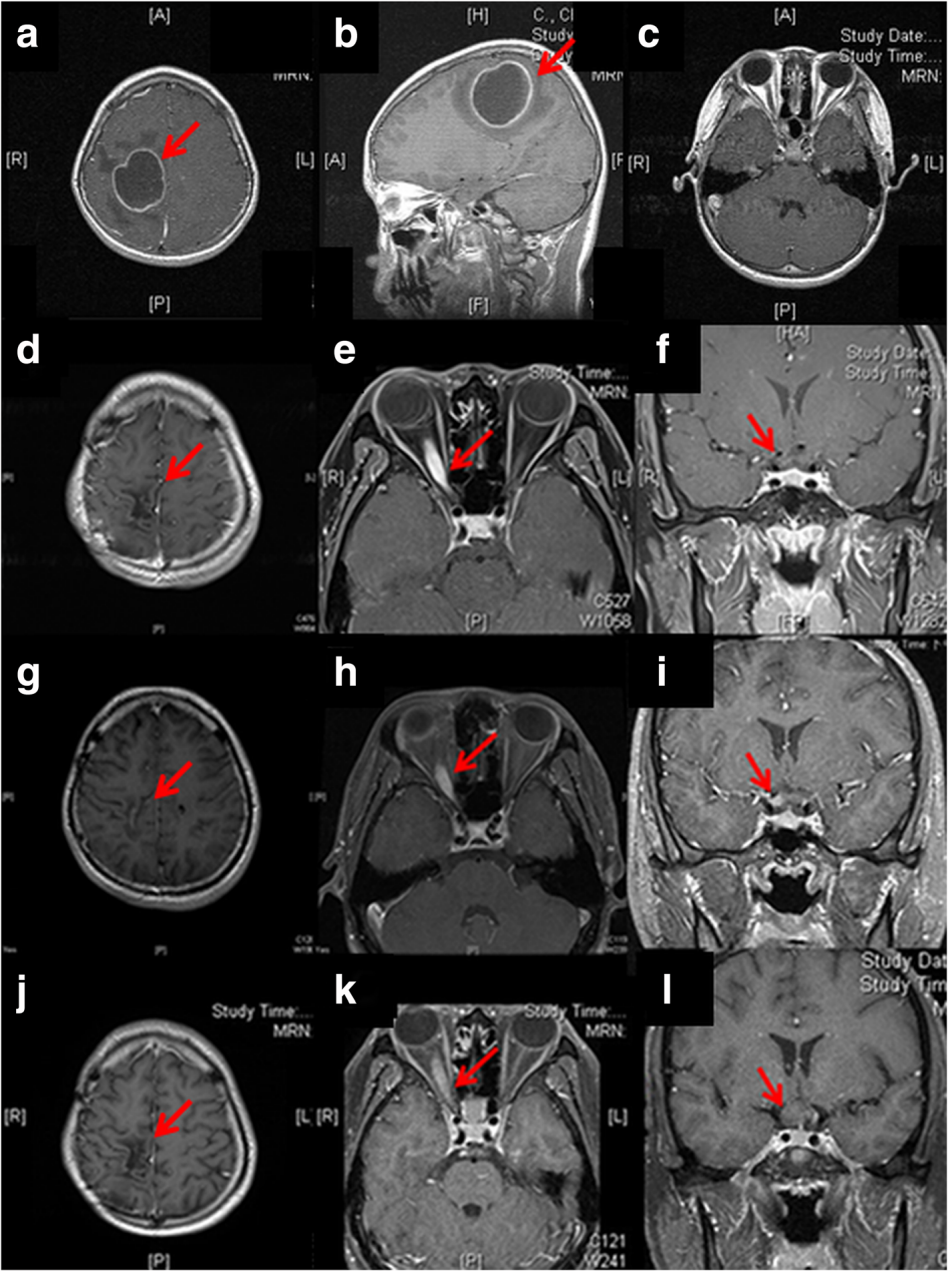
T1 weighted contrast – enhanced MRI showed (**a**, **b**) original right frontoparietal glioblastoma (**c**) without right optic nerve involvement. At the initial symptom onset, T1 weighted contrast – enhanced MRI with fat suppression showed (**d**) no evidence of brain tumor recurrence but (**e**, **f**) right pre-chaismatic enhanced fusiform optic nerve tumor. At 3 months after symptom onset, T1 weighted contrast – enhanced MRI with fat suppression showed (**h**, **i**) right fusiform enlargement and an enhanced optic nerve tumor intraorbitally extending towards the optic chiasm even (**g**) no brain tumor recurrence. At 11 months after symptom onset, T1 weighted contrast – enhanced MRI with fat suppression showed (**k**, **l**) right optic nerve tumor extending to the optic chiasm and progressing in size (**j**) without brain tumor recurrence

The 21-year old man was diagnosed with brain GBM at 13 years of age (Fig. 2a, b). After tumor extirpation, he regularly underwent temozolomide chemotherapy and radiotherapy (66.6 Gy) from February 2007 to April 2007 and no brain GBM recurrence was shown at brain MRI during this round of treatment. However, progressing in cystic size was noted during regular clinics visit after previous treatment, radical resection of tumor was done in August 2007. Then he maintained with Temozolomide treatment for 8 more years (111 cycles). In August 2015, he visited our clinic complaining of an intermittent shadow in his vision when changing positions (duration 1–2 min/attack, >5 times/day) for several years. The best-corrected visual acuity (BCVA) was 20/20 in both eyes. Ocular examinations revealed a right relative afferent pupillary defect (RAPD) and optic disc swelling (OD; Fig. 1a). Visual field (VF) testing with a Humphrey automated perimeter (Swedish Interactive Threshold Algorithm, SITA) revealed an enlarged blind spot (OD; Fig. 1c). Optical coherence tomography showed severe optic disc head swelling and normal central macular thickness. The patient received intravenous methylprednisolone for 3 days for suspected optic neuritis (OD). Orbital MRI revealed a pre-chiasmatic enhanced fusiform optic nerve tumor on a T1- weighted contrast image, typical of optic nerve glioma (Modified Dodge classification : 1aR) [9] (Fig. 2e, f). No tumor recurrence was found in brain MRI (Fig. 2d). Cerebrospinal fluid (CSF) examination revealed no definite malignant cells. Based on the diagnosis of an optic nerve tumor, likely secondary to GBM, aggressive chemotherapy (temozolomide 380 mg) was administered. Three months later, BCVA (OD) deteriorated to 20/30 and the VF was severely constricted with only central area preservation (Fig. 1d). A follow-up orbital MRI showed progressive changes in the right optic nerve lesion involving the chiasm on a T1- weighted image (Modified Dodge classification : 2cR) [9] (Fig. 2h, i). One month later, BCVA (OD) worsened to 2/200. Histopathology from a right optic nerve biopsy revealed glioblastoma. One month after the biopsy, the patient received combined concurrent chemoradiotherapy (CCRT) with temozolomide (140 mg/day) and irradiation of the involved field (60 Gy, 30 times). Afterwards, a new regimen of chemotherapy was administered (Bevacizumab) and he reported no light perception (OD). The complete clinical course is presented as time table (Table 1).

**Table 1:** Time table

	Relevant Past Medical History and Interventions		
	The 21-year old man was diagnosed with GBM at 13 years of age. After tumor extirpation, he regularly underwent temozolomide chemotherapy and radiotherapy (66.6 Gy) from February 2007 to April 2007 and no brain GBM recurrence was shown at brain MRI during this round of treatment. However, progressing in cystic size was noted during regular clinics visit after previous treatment, radical resection of tumor was done in August 2007. Then he maintained with Temozolomide treatment for 8 more years (111 cycles).		
Date	Summaries from Initial and Follow-up Visits	Diagnostic Testing (including dates)	Interventions
2015.8	Chief complaint of an intermittent shadow in the vision when changing positions for several years. Initial diagnosis: optic nerve tumor, likely secondary to GBM.	BCVA:20/20 in both eyes. RAPD(+) and optic disc swelling (OD). VF: enlarged blind spot (OD). Orbital MRI: enhanced fusiform optic nerve tumor before the optic chiasma on a T1- weighted contrast image.	Aggressive chemotherapy (temozolomide 380 mg).
2015.11	Clinical course deteriorated 3 months after symptom onset.	BCVA :20/30(OD). VF: severely constricted with only central area preservation(OD). Orbital MRI: progressive changes in the right optic nerve lesion involving the chiasm on a T1- weighted image.	
2015.12	Clinical course severely deteriorated 4 months after symptom onset. Histopathology proved malignant optic glioblastoma.	BCVA:2/200. Histopathology from a right optic nerve biopsy: glioblastoma.	One month after the biopsy: CCRT with temozolomide (140 mg/day) and irradiation of the involved field (60 Gy, 30 times). Afterwards, a new regimen of chemotherapy was administered (Bevacizumab).
2016.7	Optic nerve tumor progressed 11 months after symptom onset.	Brain MRI: a right optic nerve tumor extending to the optic chiasm with progression in size.	Underwent right optic nerve tumor resection.
2016.8	The ability to have CCRT can preserve visual function in the fellow eye.	BCVA and VF were within the normal range in the left eye and no recurrence or metastasis was noted in the latest follow-up brain MRI.	

However, at 11 months after symptom onset, brain MRI showed a right optic nerve tumor extending to the optic chiasm with progression in size (Modified Dodge classification : 2b R/L) [9] (Fig. 2k, l). He underwent a right optic nerve tumor resection.

One year after symptom onset, BCVA and VF were within the normal range in the left eye (oculus sinister, OS) and no recurrence or metastasis was noted in the latest follow-up brain MRI.

## Discussion and Conclusions

GBM is a highly malignant brain tumor with a poor prognosis. GBM constitutes approximately 17 and 6.5% of intracranial neoplasms in adult and pediatric populations, respectively [10, 11].

Currently, standard care to improve GBM patient survival begins with surgical removal followed by radiation and chemotherapy [2]. Despite optimal treatment, most patients die of the disease, and the median overall survival time is 12–15 months [1, 12]. The main parameters associated with long-term survival are younger age (<40 years), good performance status, and the ability to undergo gross total resection, followed by radiotherapy and chemotherapy [2, 10, 13]. GBM can easily metastasize within the neuroaxis (i.e., the meninges or spinal cord) via the CSF occurring in approximately 20% of GBM patients [14]. GBM metastases outside the central nervous system are rare [15], but may be found in the extraocular muscles, limbus, or orbit [16, 17]. Systemic chemotherapy is the only treatment that considerably prolongs overall survival times [12], which seem not to differ regardless of GBM developing extracranial metastasis.

The most common tumor of the optic nerve is optic nerve glioma, which represents approximately 66% of all primary optic nerve tumors. Approximately 90% of optic gliomas occur in children and most cases are relatively benign [18]. Malignant optic gliomas are rare, and mostly present in middle aged men [19]. From 1900 to 2015, only 22 cases of pathology proven optic nerve glioblastomas were recorded among 66 cases of primary malignant optic gliomas [7, 8]. At onset, 70% patients with malignant optic nerve gliomas present with symptoms of acute unilateral visual loss, spreading rapidly over the chiasm and leading to total blindness typically within 3 months [8].

Neuroradiologic findings are unspecific and typically described as contrast enhancement and thickening of the optic nerve, chiasm, or tract, with lesions having hypo- to iso-intensity on T1-weighted images [20]. Biopsies continue to be necessary to confirm a diagnosis [7].

The current standard therapy consists of surgical resection or biopsy as feasible, followed by radiotherapy and/or temozolomide chemotherapy for high grade astrocytomas [7, 21]. The prognosis of a malignant optic glioma is fatal and most patients die within 1 year [7, 8].

Our case was challenging; diagnosis was difficult before biopsy because the optic nerve tumor appeared 7 years after brain GBM and visual function was initially normal. In addition, the complications of radiotherapy were a concern. The relationship between delayed radiotherapy and the progression and extension of the optic nerve tumor was unclear. Fortunately, BCVA and VF in the fellow eye remained unchanged despite the chiasm becoming involved 1 year after the symptom onset. Compared with previous reports, the progression of the optic malignant tumor was slower and the prognosis was better.

In summary, malignant optic tumors may appear >5 years after brain GBM. Regular ophthalmic examinations and brain MRIs are recommended for early diagnosis. Additional studies should evaluate the role and optimal timing of early adjuvant radiotherapy before visual function decreases. Young age, good health, and the ability to undergo CCRT can palliate the clinical course and preserve visual function in the fellow eye.

## Abbreviations

AA: Anaplastic astrocytoma
BCVA: Best-corrected visual acuity
CCRT: Concurrent chemoradiotherapy
CSF: Cerebrospinal fluid
GBM: Glioblastoma multiforme
MRI: Magnetic resonance imaging
oculus dexter: OD, Right eye
oculus sinister: OS, Left eye
RAPD: Relative afferent pupillary defect
SITA: Swedish Interactive Threshold Algorithm
VF: Visual field
